# Extrasynaptic NMDA receptor-induced tau overexpression mediates neuronal death through suppressing survival signaling ERK phosphorylation

**DOI:** 10.1038/cddis.2016.329

**Published:** 2016-11-03

**Authors:** Xu-Ying Sun, Qing-Zhang Tuo, Zhen-Yu Liuyang, Ao-Ji Xie, Xiao-Long Feng, Xiong Yan, Mei Qiu, Shen Li, Xiu-Lian Wang, Fu-Yuan Cao, Xiao-Chuan Wang, Jian-Zhi Wang, Rong Liu

**Affiliations:** 1 Department of Pathophysiology, School of Basic Medicine and the Collaborative Innovation Center for Brain Science, Key Laboratory of Ministry of Education of China for Neurological Disorders, Tongji Medical College, Huazhong University of Science and Technology, Wuhan 430030, China; 2 Biological Engineering and Regenerative Medicine Center, Tongji Hospital, Tongji Medical College, Huazhong University of Science and Technology, Wuhan 430030, China; 3 Co-innovation Center of Neuroregeneration, Nantong 226000, China

## Abstract

Intracellular accumulation of the hyperphosphorylated tau is a pathological hallmark in the brain of Alzheimer disease. Activation of extrasynaptic NMDA receptors (E-NMDARs) induces excitatory toxicity that is involved in Alzheimer's neurodegeneration. However, the intrinsic link between E-NMDARs and the tau-induced neuronal damage remains elusive. In the present study, we showed in cultured primary cortical neurons that activation of E-NMDA receptors but not synaptic NMDA receptors dramatically increased tau mRNA and protein levels, with a simultaneous neuronal degeneration and decreased neuronal survival. Memantine, a selective antagonist of E-NMDARs, reversed E-NMDARs-induced tau overexpression. Activation of E-NMDARs in wild-type mouse brains resulted in neuron loss in hippocampus, whereas tau deletion in neuronal cultures and in the mouse brains rescued the E-NMDARs-induced neuronal death and degeneration. The E-NMDARs-induced tau overexpression was correlated with a reduced ERK phosphorylation, whereas the increased MEK activity, decreased binding and activity of ERK phosphatase to ERK, and increased ERK phosphorylation were observed in tau knockout mice. On the contrary, addition of tau proteins promoted ERK dephosphorylation *in vitro*. Taking together, these results indicate that tau overexpression mediates the excitatory toxicity induced by E-NMDAR activation through inhibiting ERK phosphorylation.

Hyperphosphorylated tau is the major component of neurofibrillary tangles (NFTs) in Alzheimer disease (AD). The levels of total tau are about eight-fold higher in AD than in control cases, and this increase is mainly in the form of the abnormally phosphorylated protein.^1, 2^ Increased level of tau mRNA is also present in AD cases.^3, 4^ Tau protein is essential for microtubule assembly and microtubules stabilization. However, tau overexpression makes itself easier to be aggregated, phosphorylated and ultimately promotes NFTs formation.^5^ Furthermore, excessive tau could also accelerate neuronal degeneration in several ways: studies show tau interacting with post-synaptic signaling complexes, disturbing the trafficking or targeting of synaptic receptors, influencing transport and function of synaptic mitochondria, and mediating the synaptotoxicity induced by *β*-amyloid.^6^ Thus, tau overexpression has an important role in AD development, but till now the upstream factors inducing tau overexpression and accumulation are elusive.

N-methyl-d aspartate receptor (NMDAR) is a cation channel, which is gated by the neurotransmitter glutamate. Physiological activation of NMDAR plays key roles in multiple neurological functions such as synaptic plasticity, neuronal development and survival, learning and memory.^7^ On the contrary, excessive NMDAR activation is toxic to neurons. The mechanisms for the bipolar effects of NMDAR are widely studied by researchers. Hardingham *et al.*^8^ reported that different effects of NMDARs were determined by their synaptic or extrasynaptic localization. Activation of synaptic NMDAR is suggested to improve synaptic plasticity and learning and memory ability, promote neuronal survive and maturation; while activation of extrasynaptic NMDAR (E-NMDAR) could induce neuronal death, synaptic plasticity failure and memory loss,^8^ thus contribute to phenotype onset in Huntington's disease, stroke and AD.^9, 10, 11^ The downstream signaling pathways leading to neuronal survival by synaptic NMDAR and neuronal death by E-NMDAR are widely investigated. Extracellular signal-regulated kinase 1/2 (ERK1/2) signaling has a key role in cell survival.^8, 12^ Leveille *et al.*^12^ reported that selective synaptic NMDAR activation induced ERK activation, whereas E-NMDAR activation could not, indicating that E-NMDAR activation shuts off ERK signaling pathway. The underlying mechanism for ERK signaling shutting off by E-NMDARs remains unclarified.

A recent study showed chronic activation of E-NMDARs promoted amyloidogenic KPI-APP expression leading to neuronal A*β* release, representing a pathological mechanism of E-NMDARs in AD development.^11^ Another study suggested a toxic exposure to glutamate enhances tau mRNA expression in primary neuronal cultures.^13^ However, as glutamate incubation activates both the synaptic and extrasynaptic NMDA receptors, the role of E-NMDARs in this process was not distinguished. In the present study, we explored the effect of E-NMDAR activation on tau expression and its role in neurodegeneration. We found that selective extrasynaptic but not synaptic NMDA receptor activation induced tau overexpression and neuronal degeneration/death in cultured primary neurons and mouse brain hippocampus, which could be reversed by pretreatment of memantine, an antagonist of E-NMDARs. In tau knockout (Ko) mice or neurons, selective activation of E-NMDARs failed to induce cell death, with retained surviving signaling ERK activation. Increased mitogen-activated and extracellular signal-regulated kinase kinase (MEK) activity, decreased binding and activity of ERK phosphatase to ERK, and increased ERK phosphorylation was observed in tau Ko mice, whereas addition of tau proteins into tau Ko mice brain homogenates promoted the ERK dephosphorylation *in vitro*. Thus, overexpressed tau may mediate the toxicity of extrasynaptic NMDA receptors through depressing survival signaling ERK phosphorylation.

## Results

### Activation of extrasynaptic but not synaptic NMDA receptors increases tau protein expression with neurodegeneration in cultured cortical neurons

#### Activation of E-NMDARs increases tau protein level in rat cortical neurons

**Figure 1:**
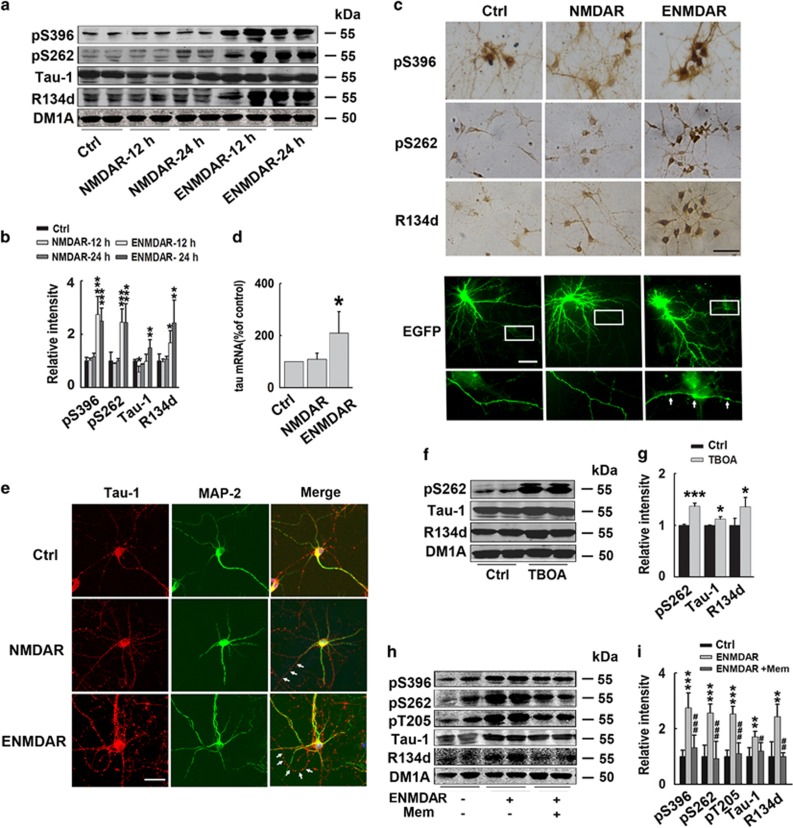
Activation of extrasynaptic but not synaptic NMDA receptors increases tau expression with neurodegeneration in cortical neurons. (**a**) Rat primary cortical neurons (12–14 DIV) were incubated with Bic (50 *μ*M) /4-AP (2.5 mM) to activate synaptic NMDA receptors for 12 or 24 h (herein used as NMDAR). To specifically induce extrasynaptic NMDA receptors (E-NMDAR) activation, neurons were incubated with Bic (50 *μ*M)/4-AP (2.5 mM) for 2 min, after wash, open NMDA receptors blocker MK-801 (10 *μ*M) was administrated for another 2 min to block synaptic NMDA receptors, at last NMDA (30 *μ*M) and glycine (10 *μ*M) were used to selectively activate E-NMDARs for 12 or 24 h (herein used as E-NMDAR). Total tau and phosphorylated tau at Ser396 and Ser262 sites, and dephosphorylated tau level at Tau-1(Ser198/199/202) sites were detected by western blotting. (**b**) Quantitative analysis of the blots in (**a**). Total, phosphorylated and dephosphorylated tau levels were normalized with DM1A. *P<0.05, **P<0.01 and ***P<0.001 *versus* control neurons, *n*=8, *N*=4 independent cultures. (**c**) Upper panel: primary cortical rat neurons (12–14 DIV) were treated with synaptic or extrasynaptic NMDA receptors activation protocols as previously described for 24 h. Images from DAB immunocytochemistry staining with pS396, pS262, R134d (total tau) antibodies were acquired under a confocal microscope. Scale bar=100 *μ*m; Bottom panel: primary cortical rat neurons (11 DIV) were transfected with surface EGFP to visualize the neuronal morphology, 48 h later, neurons were treated with synaptic or extrasynaptic NMDAR activation protocols for 24 h. At the end of treatment, cells were fixed with 4% paraformaldehyde and observed under the fluorescence microscope. Amplified images of axons in the rectangle were showed at the bottom. White arrows showed puncta-like enhanced EGFP fluorescence in the axon. Scale bar=20 *μ*m. (**d**) Primary cortical rat neurons (12–14 DIV) were treated with synaptic or extrasynaptic NMDA receptors activation protocols for 6 h, total RNA was extracted from neuronal cultures. Real-time PCR analysis was performed to quantify relative expression of tau mRNA in the different groups. The expression level of *tau* gene was analyzed according to the Ct method (comparative Ct method), in which Ct is the threshold cycle value and normalized by *β*-actin. Histograms represent means±S.D., and statistical analysis was performed by ANOVA (*n*=6, *N*=3 independent cultures; *P<0.05 *versus* control). (**e**) Primary cortical mouse neurons (12–14 DIV) were treated with synaptic or extrasynaptic NMDA receptors activation protocols for 24 h, immunofluorescence staining images with Tau-1 (red) and MAP-2 (dendrite marker, green) were acquired under a confocal microscope. Scale bar=50 *μ*m. White arrows showed the axons that were only stained by Tau-1 antibody. (**f**) Rat primary cortical neurons (12–14 DIV) were incubated with Bic/4-AP for 2 min to activate synaptic NMDA receptors, then incubated with NMDA receptors blocker MK-801 (10 *μ*M) for another 2 min to block synaptic NMDAR, at last glutamate transporters blocker DL-TBOA (30 *μ*M) was used to selectively induce extrasynaptic NMDA receptors activation for 24 h. Total (R134d), phosphorylated (pS262) and dephosphorylated tau (Tau-1) levels were detected by western blotting. (**g**) Quantitative analysis of the blots in (**f**). Total, phosphorylated and dephosphorylated tau levels were normalized with DM1A. *P<0.05, ***P<0.001 *versus* control group, *n*=6, *N*=3 independent cultures. (**h**) Rat primary cortical neurons were treated as previously described to activate E-NMDARs for 24 h, with or without pretreatment of extrasynaptic NMDA receptor antagonist memantine (1 *μ*M) for 30 min, total tau and phosphorylated tau levels at Ser396, Ser262 and Thr205, and dephosphorylated tau level at Tau-1 (Ser198/199/202) sites were detected by western blotting. (**i**) Quantitative analysis of the blots in (**h**). Total, phosphorylated and dephosphorylated tau levels were normalized with DM1A. **P<0.01, ***P<0.001 *versus* control group; ^#^*P*<0.05, ^##^*P*<0.01 and ^###^*P*<0.001 *versus* extrasynaptic NMDAR activation group, *n*=6, *N*=3 independent cultures

To evaluate the effect of E-NMDAR activation on tau expression, we first performed the experiments on cultured rat cortical neurons at DIV 12–14 with mature dendrites and axons. Selective synaptic NMDAR stimulation was achieved by blocking GABA_A_ receptor with 50 *μ*M bicucullin (Bic) in the presence of 4-aminopyridine (4-AP) (2.5 mM), a weak potassium channel blocker, herein used as NMDAR.^14^ For E-NMDAR stimulation, we first activated synaptic NMDAR by applying Bic/4-AP treatment, after having blocked activated synaptic NMDAR with 10 *μ*M MK-801, 30 *μ*M NMDA and 10 *μ*M glycine were applied to selectively activate E-NMDAR,^12^ herein named E-NMDAR treatment. The activation of synaptic NMDAR or E-NMDAR was confirmed by increased brain-derived neurotrophic factor (BDNF) expression or retarded CAMP response element binding protein (CREB) phosphorylation, respectively (Supplementary Figure S1). The results showed that synaptic NMDAR activation for 12  and 24 h did not change total (R134d) and phosphorylated (pS396, pS262) tau levels, except that Tau-1-recognized (Ser198/199/202 dephosphorylated) tau was slightly decreased at 12 h. While E-NMDAR activation for 12 and 24 h resulted in significant increase of total (R134d), phosphorylated (pS396, pS262) and dephosphorylated (Tau-1) tau. At the end of treatment, total tau level increased by about two-fold, with Ser396 and Ser262 hyperphosphorylated tau increased by about 2.5 times, Ser198/199/202 dephosphorylated tau increased by 1.5 times (Figures 1a and b). We further explored the phosphorylation level of tau by calculating the ratio of phosphorylated /dephosphorylated tau to total tau. The result showed that E-NMDAR activation only slightly increased tau phosphorylation levels (Supplementary Figure S2), with no significant difference compared with the control group. The increase of phosphorylated tau levels was due to increase of total tau. This result indicated that E-NMDAR activation mainly induces overexpression but not hyperphosphorylation of tau. Diaminobenzidine (DAB) staining with pS396, pS262 and R134d further confirmed the enhancement of tau expression induced by E-NMDAR activation (Figure 1c). To confirm the specific effect of E-NMDARs on tau, coapplication of nimodipine (L-type voltage sensitive Ca^2+^ channels blocker) or CNQX (AMPA receptors antagonist) was performed in E-NMDAR activation protocol. The results showed that neither nimodipine nor CNQX could block tau overexpression, confirming the effect of E-NMDA receptors on increasing tau levels (Supplementary Figure S3). Anterograde organelle transport was known to be interrupted by tau overexpression in cultured cells.^15^ To detect the change of neurites, we transfected surface-enhanced green fluorescent protein (Suf-EGFP) to show the cell morphology. In E-NMDAR-activated neurons, puncta-like-enhanced EGFP fluorescence was observed in axon, indicating a disrupted axonal transport. At the same time, number of dendrites was decreased, with shortened dendrite length (Figure 1c). The morphology of the neurons suggests that E-NMDAR activation have induced degeneration of both axon and dendrites. Thus, activation of extrasynaptic NMDA receptors increases tau protein expression and induces neurodegeneration in primary rat cortical neurons.

#### Activation of E-NMDARs increases tau mRNA level in rat cortical neurons

To test whether the modifications of tau expression were altered at transcriptional level, we detected tau mRNA level by real-time PCR. The results showed that activation of extrasynaptic but not synaptic NMDAR for 6 h increased tau mRNA level by about two-fold (Figure 1d), indicating that the increased transcription of tau contributes to the E-NMDAR activation-induced tau overexpression.

#### Activation of E-NMDARs increases tau expression with neurodegeneration in mouse cortical neurons

We also confirmed the effect of E-NMDAR on tau expression in cultured mouse primary cortical neurons. By immunofluorescence staining of Tau-1 and dendrite marker MAP-2, the degeneration-associated axonal puncta staining of Tau-1 was observed. Furthermore, increased tau also translocalized to and accumulated in dendrites (Figure 1e).

#### Treatment with glutamate transporters blocker DL-TBOA increases tau protein level in rat cortical neurons

To further validate the effect of E-NMDAR activation on tau expression, we used another procedure to specifically induce E-NMDAR activation in rat cortical neurons. Dl-threo-b-benzyloxyaspartic acid (DL-TBOA) is the competitive blocker of the glutamate transporter; its application could cause glutamate spillover to extrasynaptic field and indirectly activate E-NMDARs.^15, 16^ In our experiments, DL-TBOA (30 *μ*M) treatment for 24 h also significantly increased the levels of total (R134d), phosphorylated (pS262) and dephosphorylated tau proteins (Tau-1) (Figures 1f and g).

#### Memantine reverses E-NMDARs activation-induced tau overexpression in rat cortical neurons

Memantine is an uncompetitive, low-affinity, open NMDA receptor channel blocker; it is thought to selectively block the E-NMDA receptors but does not interfere with normal synaptic transmission.^17^ To confirm the effect of E-NMDAR activation on tau expression, we used memantine treatment to see whether it can reverse E-NMDAR activation-induced tau overexpression. Rat primary cortical neurons were pre-incubated with memantine (1 *μ*M) for 30 min, and then E-NMDA receptors were activated as previously described. E-NMDAR activation caused tau overexpression as fore-mentioned, whereas memantine pretreatment significantly rescued E-NMDAR activation-induced elevation of total, phosphorylated and dephosphorylated tau, the three forms of tau protein were almost recovered to control levels (Figures 1h and i). These results further confirmed that E-NMDA receptors activation increases tau expression.

### Tau deletion protects neurons from E-NMDAR-triggered neuronal death and degeneration in neuronal cultures and mouse hippocampus

**Figure 2:**
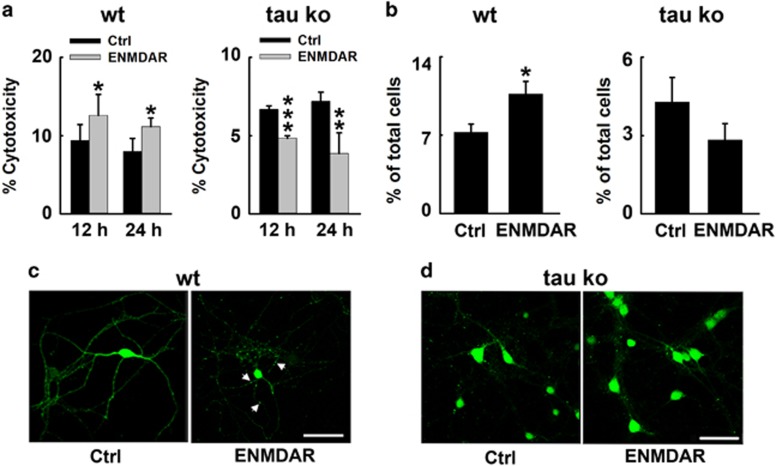
Tau deletion protects neurons from E-NMDAR-triggered neuronal death and degeneration in cortical neurons. (**a**) Primary cultured mouse cortical neurons at DIV 12–14 were subjected to extrasynaptic NMDAR activation for 12 or 24 h. The level of LDH released into the culture medium was determined by measuring the decrease in absorbance at 490 nm resulting from the oxidation of NADH. LDH levels in wildtype (Wt, left) or tau knockout (tau Ko, right) neurons treated with DMSO (Ctrl) or extrasynaptic NMDAR activation protocols (E-NMDAR) during the indicated times (12 or 24 h). *P<0.05, **P<0.01 and ***P<0.001 *versus* control group, *n*=20, *N*=3 independent cultures. (**b**) Quantification of apoptotic cells in Wt (left) or tau Ko (right) neuron cultures exposed to extrasynaptic treatment for 24 h. Quantification of the cell populations was achieved on four independent cultures. About 6000 cells were evaluated in each group. *P<0.05 *versus* control group. (**c**) Wt mouse primary cortical neurons at 5 DIV were transfected with EGFP by lentivirus. At 12 DIV, neurons were subjected to extrasynaptic NMDAR activation for 24 h. Morphological changes of EGFP-labeled neurons treated with DMSO (Ctrl) or the extrasynaptic NMDA receptor activating protocol for 24 h (E-NMDAR). Images were acquired by confocal microscopy. White arrows showed abnormal neurodegeneration. (**d**) Representative neuron images from tau Ko mouse cortical neurons treated with DMSO (Ctrl) or E-NMDARs activation protocol (E-NMDAR) for 24 h, neurons were directly fixed and visualized under the fluorescence microscope. Scale bar=50 *μ*m

We have observed that E-NMDAR activation induced tau overexpression, together with neuronal degeneration. To further explore the role of E-NMDAR activation-induced tau overexpression in neurodegeneration, we detected the effect of E-NMDAR activation on neuronal survival both in wild-type and tau-deleted primary mouse neurons and brains. We measured lactate dehydrogenase (LDH) release in the bath medium at 12 and 24 h after the onset of extrasynaptic NMDAR stimulation protocol both in wild-type and tau Ko primary mouse cortical neurons. A significant increase in LDH release after E-NMDAR exposure for 12 or 24 h in wild-type mouse neurons was observed. Surprisingly, in tau Ko mouse neurons, extrasynaptic NMDAR activation decreased LDH release, which indicating that extrasynaptic NMDAR stimulation does not have obvious cytotoxicity when tau is knockout (Figure 2a). We also counted the apoptotic cell numbers in different groups; the results showed that in wild-type mouse neurons, E-NMDAR activation triggered neuronal apoptosis, whereas the phenomenon was not observed in tau-deleted neurons (Figure 2b). We then infected cultured mouse primary neurons with lentivirus-EGFP to label the neurons at DIV5, 7 days later, the neurons were treated with E-NMDAR activation protocol. The survival neurons showed varicosities and inhomogenous EGFP imaging in the neurites (Figure 2c). These data indicated that E-NMDAR activation caused neuronal death and degeneration. To further confirm the role of tau in E-NMDAR activation-induced cell death, we cultured primary neurons from tau Ko mice. These neurons were genetically EGFP knocked in,^18^ thus tau-deleted neurons were all labeled with EGFP. The morphology of the neurons showed no change after E-NMDAR activation (Figure 2d). These results suggested that tau deletion prevented neurons from E-NMDAR -induced cell death and degeneration.

**Figure 3:**
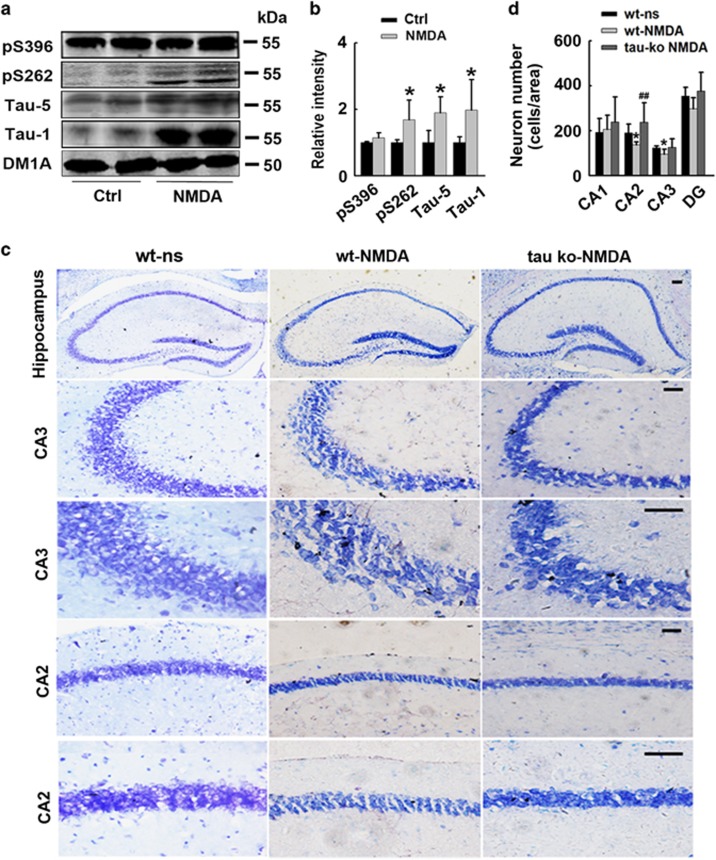
Tau deletion protects neurons from E-NMDARs-triggered neuronal death in mouse hippocampus. (**a**) Wild-type (Wt) C57 mice were injected with saline (Ctrl) or NMDA (60 mM, 2 *μ*l) into the hippocampus, 24 h later, hippocampi were isolated and homogenized, total tau (Tau-5), dephosphorylated tau levels at Tau-1 (Ser198/199/202) sites and phosphorylated tau levels at Ser396 and Ser262 sites were detected by western blotting. (**b**) Quantitative analysis of the blots in (**a**). Total, phosphorylated and dephosphorylated tau levels were normalized with DM1A. *P<0.05 *versus* saline-injected control mice, *n*=3 per group. (**c**) Wt or tau Ko mice were injected with saline (NS) or NMDA (60 mM, 2 *μ*l) into the hippocampus. Twenty-four hours later, half brain of the mice was fixed. Neurons in the hippocampus were stained by Nissl staining. Representative images from the hippocampus, scale bars=50 *μ*m. (**d**) Quantitative analysis of the neuron number in CA1, CA2, CA3 and DG regions of hippocampus, *P<0.05 *versus* Wt NS group. ^##^*P*<0.01 *versus* NMDA-treated Wt mice (*n*=3 mice per group, cells in 10 brain slices were counted for each animal)

Although the extrasynaptic NMDAR activation protocol in animal brains is experimentally unachievable, several studies have described a rapid modification of cerebral gene expression (at both mRNA and protein levels) in mice subjected to intraperitoneal NMDA injection.^19, 20^ To validate our *in vitro* findings, we injected NMDA into the mouse hippocampus directly to induce extensive activation of NMDA receptors, including extrasynaptic NMDA receptor. We first evaluated the expression of tau after NMDA injection, the result showed a significant increase of total (Tau-5), phosphorylated (pS262) and dephosphorylated (Tau-1) tau levels in mouse brains compared with the saline-injected control group (Figures 3a and b), which was consistent with the changes observed in cultured primary neurons treated with E-NMDAR activation protocol. We then used Nissl staining to detect neuron survival of the three groups. Results showed NMDA injection induced significant neuron loss in CA2 and CA3 regions of the hippocampus of wild-type mice; whereas in tau Ko mice, NMDA injection did not reduce neuronal survival (Figures 3c and d).These *in vivo* results reinforced the idea that E-NMDAR activation triggers tau expression, and increased tau could promote neuronal degeneration and death.

### Tau deletion restores ERK activation in E-NMDAR-activated mouse cortical neuron cultures and hippocampus

**Figure 4:**
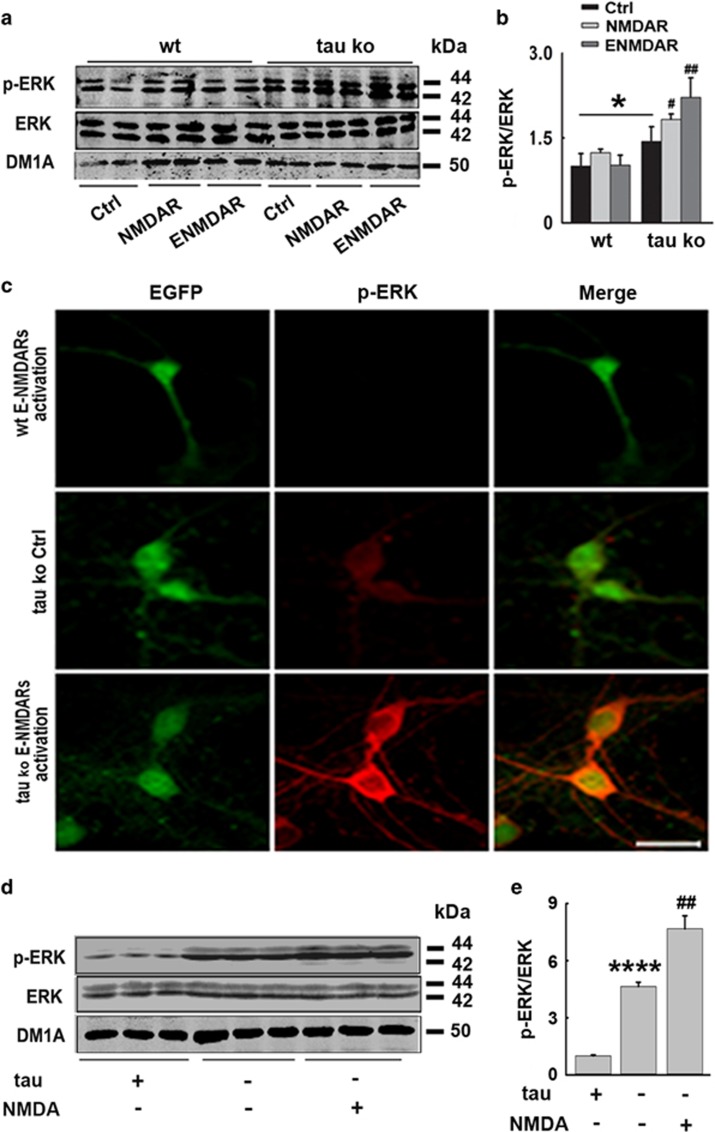
Tau deletion restores ERK activation in E-NMDAR-activated primary mouse cortical neurons and hippocampus. (**a**) Wt- or tau-deleted mouse neurons were cultured and treated with synaptic or extrasynaptic NMDAR activating protocols for 24 h, respectively. The total protein levels and active forms of ERK were detected by western blotting. (**b**) Quantitative analysis of the protein levels in (**a**), *P<0.05 *versus* wild-type neurons, ^#^*P*<0.05 *versus* tau Ko control neurons, ^##^*P*<0.01 *versus* tau Ko control neurons, *n*=6, *N*=3 independent cultures. (**c**) Wt or tau Ko mouse neurons were cultured and treated with or without E-NMDAR-activating protocols for 24 h, p-ERK and EGFP staining were acquired by confocal microscopy, for Wt neurons, the cells were transfected with EGFP lentivirus to be visualized. Scale bar=50 *μ*m. (**d**) Wt or tau Ko mice were injected with saline (NS) or NMDA (60 mM, 2 *μ*l) into the hippocampus. Twenty-four hours later, left part of hippocampi was isolated and homogenized, total protein levels and active forms of ERK were detected by western blotting. (**e**) Quantitative analysis of the ERK levels in (**d**), ****P<0.0001 *versus* Wt neurons, ^##^*P*<0.01 *versus* tau Ko control neurons, *n*=3 mice per group

To further explore the underlying mechanisms for the role of increased tau in E-NMDAR activation-induced neuronal degeneration and death, we measured the ERK signaling activity by detecting the ERK1/2 phosphorylation levels both in E-NMDAR-activated primary mouse cortical neurons and hippocampus. The results indicated that in cultured wild-type neurons, E-NMDA receptor activation could not induce ERK phosphorylation, which was consistent with that was previously reported,^12^ whereas in primary neurons from tau Ko mice, ERK showed obviously higher phosphorylation level than that in wild-type neurons. After E-NMDAR activation, ERK phosphorylation was further robustly increased, which was even higher than that in synaptic NMDA receptor activation (Figures 4a and b). Immunofluorescence staining further reinforced this result; E-NMDAR activation did not induce ERK phosphorylation in wild-type neurons, but induced strong ERK activation in tau-deleted neurons. Staining of phosphorylated ERK was mainly observed in the cytoplasm and neurites, with less located in nucleus (Figure 4c).

E-NMDAR activation was also induced by NMDA injection into the mouse hippocampus directly as previously described. Similarly, higher basal ERK phosphorylation level was observed in tau deletion mouse brains, whereas E-NMDAR activation in these mice resulted in retained, strong ERK phosphorylation (Figure 4d). Thus, tau deletion rescued ERK signaling activation when E-NMDARs were selectively activated, indicating that tau suppresses ERK signaling pathway in neurons.

### Tau protein suppresses ERK signaling pathway through inhibiting MEK and promoting ERK dephosphorylation by recruiting ERK phosphatase

**Figure 5:**
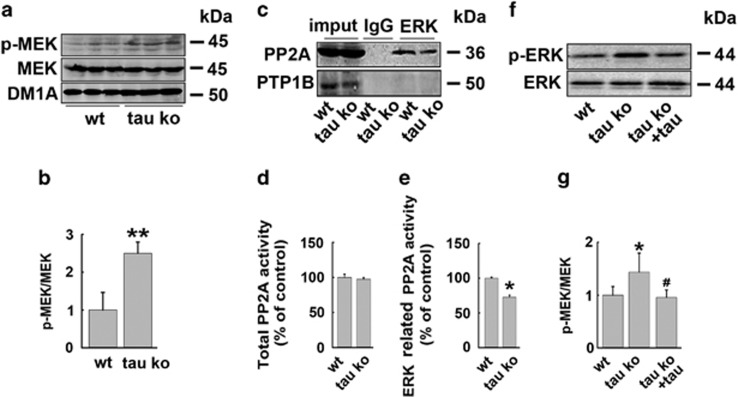
Tau protein suppresses ERK signaling pathway through inhibiting MEK and promoting ERK dephosphorylation by recruiting ERK phosphatase. (**a**) Wt and tau Ko mice hippocampi were homogenized. The total protein levels and active forms of MEK were detected by western blotting. (**b**) Quantitative analysis of the p-MEK level in (**a**), **P<0.01 *versus* Wt mice, *n*=3 mice per group. (**c**) Wt and tau Ko mice hippocampal lysates were immunoprecipitated with anti-ERK antibody, then detected with anti-PP2A and PTP1B antibodies through western blotting. *n*=3 mice per group. (**d**) Wt and tau Ko mice hippocampi were homogenized and subjected to PP2A activity assay, *n*=3 mice per group. (**e**) Wt and tau Ko mice hippocampal lysates were co-immunoprecipitated with anti-ERK antibody, and then were subjected to PP2A activity assay, *P<0.05 *versus* Wt mice, *n*=3 mice per group. (**f**) His-tagged phosphorylated ERK protein was incubated for 10 min with Wt or tau Ko hippocampal homogenates in the presence of U0126 (10 *μ*M), with or without the addition of recombinant human tau441 protein. Then anti-his antibody and protein G were added to the lysates overnight at 4 °C. The phosphorylated p-ERK and total ERK were analyzed by western blotting. (**g**) Quantitative analysis of the ERK phosphorylation levels in (**f**), *P<0.05 *versus* Wt mouse brain homogenates, ^#^*P*<0.05 *versus* tau Ko mouse brain homogenates without tau441 protein addition, *n*=5 mouse hippocampi per group

To disclose the mechanisms for the suppressing effect of tau on ERK phosphorylation, we first detected the ERK kinase (MEK) activity in wild-type and tau Ko mouse hippocampus. Significantly higher MEK phosphorylation level was observed in tau Ko mice (Figures 5a and b), indicating that the existence of tau protein inhibits the activity of ERK kinase. Next, we explored whether tau could promote ERK dephosphorylation. Protein phosphatase 2A (PP2A) is one of the most important ERK phosphatase,^21^ which is also involved in MEK dephosphorylation.^22^ We detected the interaction of PP2A with ERK in wild-type and tau Ko mouse brains by immunoprecipitation. The result showed that tau deletion reduced the binding of PP2A to ERK remarkably. Protein tyrosine phosphatase-1B (PTP1B), another common tyrosine phosphatase, did not bind to ERK and was used as a negative control (Figure 5c). These data suggested that tau may promote ERK dephosphorylation by PP2A through increasing the interaction of these two proteins. This hypothesis was identified by PP2A activity assay results, which showed that the ERK-related PP2A activity was decreased in tau Ko mice although total PP2A activity was not changed (Figures 5d and e). To further validate the effect of tau in promoting ERK dephosphorylation, recombinated ERK proteins were phosphorylated *in vitro*, then the proteins were incubated with the brain homogenates from wild-type or tau Ko mice hippocampi, with the addition of MEK inhibitor U0126. In this system, the ERK dephosphorylation by phosphatases could be specifically observed and compared. The result showed that p-ERK proteins incubated with tau Ko mouse homogenates were less dephosphorylated compared with that incubated with wild-type mouse homogenates, whereas the addition of tau protein restored ERK dephosphorylation (Figures 5f and g). These data strongly indicated that tau protein promoted ERK dephosphorylation through recruiting ERK phosphatase.

## Discussion

The toxicity of tau protein has been widely recognized in neurodegenerative disease.^23^ Tau hyperphosphorylation and accumulation is an early event in the development of AD, total tau levels are about eight-fold higher in AD brain than in control cases.^1, 2^ However, the environmental factors leading to tau overexpression are not fully understood. Extrasynaptic NMDAR activation causes neuronal death and neurodegeneration,^9, 10, 11^ which is suggested to be involved in AD pathogenesis. In the present study, we explored the effect of E-NMDAR activation on tau expression both in primary neurons and in animal models, and further investigated the underlying molecular mechanisms of the neurodegeneration mediated by E-NMDAR-induced tau overexpression.

We first investigated the effect of E-NMDAR activation on tau expression in cultured primary rat and mouse cortical neurons. Selective activation of extrasynaptic but not synaptic NMDAR resulted in dramatically increased tau levels, which mimic the changes in AD brains. DL-TBOA, a competitive blocker of the glutamate transporter, whose application induces glutamate spillover to extrasynaptic site, thus indirectly activates extrasynaptic NMDAR, also induced tau overexpression. Furthermore, hippocampal NMDA injection that results in E-NMDAR activation also increased tau level in mouse brains; these *in vivo* data validate our *in vitro* results. Tau mRNA detection result showed that increased tau protein level was due to increased gene transcription. A previous study by Esclaire *et al.*^13^ also showed dramatically enhanced tau mRNA level upon glutamate incubation in cultured rat cortical neurons, though the effects of synaptic and extrasynaptic NMDA receptors were not distinguished. Our data suggested that only E-NMDAR activation could induce tau overexpression. This hypothesis was further identified by the rescue effect of memantine on tau overexpression. Memantine has been shown to preferentially block excessive NMDAR activity without disrupting normal synaptic activity.^24, 25, 26^ It is interesting to note that memantine, in the concentration (1 *μ*M) used in our experiment also inhibited Aβ production which was induced by E-NMDAR activation.^11^ Thus, E-NMDAR activation promote the development of both tauopathy and *β*-amyloidosis, and memantine may be an effective therapeutic strategy against these damages.

The underlying mechanisms of E-NMDAR activation-induced tau overexpression need further exploration. Several studies have reported a rapid modification of cerebral gene expression (at both mRNA and protein levels) in mice or neurons upon E-NMDAR activation.^11, 19, 20^ Our data indicate that tau gene expression is also modified by E-NMDA receptor. Recently, Liu *et al.*^27^ reported that tau gene transcription and expression was downregulated by PKA-CREB signaling, whereas E-NMDAR activated a general and dominant CREB shut off pathway that blocked induction of BDNF expression.^28^ Thus, E-NMDAR activation may also activate tau expression through shutting off CREB signaling.

We tried to explore the role of tau overexpression in E-NMDAR activation-induced neuronal degeneration and death. Tau hyperphosphorylation and accumulation were long-term thought to be harmful to the neurons,^23^ however, in our previous study, we found that tau may serve as a victim substrate of phosphorylation, thus protect the neurons to escape from an acute apoptotic death.^29^ So we are curious on what a role of the increased tau played in E-NMDAR activation-induced cell damage in the present study. We first observed the degeneration of neurites in rat and mouse neurons treated by E-NMDAR activation; this phenomenon was not observed in neurons treated by synaptic NMDAR activation without tau overexpression or in tau Ko neurons. Furthermore, activating of E-NMDAR for 24 h resulted in significant neuron loss both in cell cultures and in mouse brains, which could be rescued by tau deletion. These results indicate that tau overexpression accelerates neuronal degeneration and death during E-NMDAR activation. The discrepancy between the findings of present study and our previous research is possibly due to different experimental models. In the previous study, we focused to explore the role of tau phosphorylation in acute apoptotic cell death, and concluded that tau may competitively be phosphorylated as a victim instead of cell survival signal protein *β*-catenin, thus protect the cells from apoptosis.^29^ While in the present study, E-NMDAR activation mainly resulted in tau overexpression but not tau hyperphosphorylation. We suspected that a temporary phosphorylation of tau may prevent the neurons from acute apoptotic death, but long-term tau accumulation will promote the cell undergo chronic degeneration and death.

The involvement of tau protein in excitotoxicity of many neurotoxins such as A*β*, kainic acid and pentylenetetrazole has been previously reported,^30, 31, 32^ which was consistent with our findings in the present study. The question is how does tau mediate the excitotoxicity? Ittner *et al.*^33^ suggested that dendritic tau played a key role in post-synaptic targeting of Fyn, the latter, mediated the interaction of NMDA receptors and PSDs, and enhanced the cytotoxicity. Their study disclosed a mechanism of tau in mediating cytotoxicity in synapse. Then what is the mechanism of tau in mediating the toxicity of extrasynaptic NMDARs? ERK signaling has a key role in cell survival.^12^ Distinct different patterns of ERK signaling downstreamed synaptic and extrasynaptic NMDAR activation. Excitation of synaptic NMDAR induced a sustained ERK activation, whereas excitation of E-NMDAR shut off ERK activation. In the present study, we were surprised to observe a robust phosphorylation of ERK upon specific E-NMDAR excitation in tau Ko neurons and mouse brains. Even in resting state without NMDA receptor excitation, tau-deleted neurons and brain tissues also showed higher ERK phosphorylation levels than controls. These data strongly indicated that tau mediated ERK signaling shut off. Our study further indicated that tau protein could inhibit the activity of ERK kinase MEK, and promote ERK dephosphorylation by PP2A through recruiting this phosphatase to ERK. The *in vitro* dephosphorylation experiment showed delayed and inhibited dephosphorylation of p-ERK in tau Ko mouse brain homogenate, which was reversed by the addition of tau protein. Thus, tau protein has the ability to suppress ERK phosphorylation. In wild-type mouse brains and neurons, E-NMDARs activation induced tau overexpression, which shut off ERK signaling; in tau-deleted neurons, the inhibitory effect of tau on ERK activation was removed, so E-NMDARs activation could induce strong ERK phosphorylation and better cell survival, as a compensatory protective response upon the damage. Contrary to our findings in the present study, Amadoro *et al.*^34^ reported that exogenous overexpressed human tau in cultured neurons induced NMDA-NR2B receptor-dependent cytotoxicity partially through ERK activation. ERK activation has a dual role both in neuronal survival and death. Some stimulations such as BDNF, epidermal growth factor (EGF), nerve growth factor, as well as synaptic activity could induce ERK activation (which were transient in most cases), thus promote neuron growth and survival; while damage factors such as oxidants, overactivation of EGF, *β*-amyloid could induce sustained ERK activation and cell death.^35^ We suspected that exogenous overexpression of human tau in rat neurons was much stressful to neurons compared with endogenous-induced tau. Accumulation of exogenous tau may interact with more signal proteins other than PP2A, thus initiate prodeath-sustained ERK activation.

In our study, the underlying mechanism for tau inhibition on MEK needs further exploration. It is also possible that phosphatases other than PP2A are also involved in tau-mediated ERK dephosphorylation.

In summary, we have demonstrated in the present study that a prolonged E-NMDAR activation induces tau overexpression, the latter, shuts off ERK signaling through inhibiting ERK phosphorylation, and thus mediates the toxicity of the E-NMDAR activation.

## Materials and Methods

### Antibodies and reagents

Rabbit polyclonal antibodies (pAb) pS396, pS262 and pT205 (1:1000 for western blotting and 1:200 for immunohistochemistry) against tau phosphorylated at corresponding sites were from Signalway Antibody (Pearland, TX, USA). PAb of MAP-2 (1:200), mouse monoclonal antibody (mAb) Tau-1 against dephosphorylated tau at Ser198/199/202 sites (1:1000 for western blotting and 1:200 for immunohistochemistry) and Tau-5 against total tau (1:1000), mAb against His-tag, His-tagged recombinant ERK protein and recombinant human Tau441 protein were all purchased from Millipore (Billerica, MA, USA). PAbs against p-ERK (1:1000 for western blotting and 1:200 for immunohistochemistry), ERK (1:1000), p-MEK (1:1000) and mAbs against PP2A (1:1000) and MEK (1:1000) were obtained from Cell Signaling Technology (Beverly, MA, USA). PAb against PTP1B (1:1000) was from Abcam (Cambridge, UK). Protein G was from Cwbiotech Company (Beijing, China). N-Methyl-d-aspartate (NMDA, 30 *μ*M for cell culture and 2 *μ*l (60 mM) for mice hippocampal injection), Bic (50 *μ*M), 4-AP (250 *μ*M), MK-801 (10 *μ*M) and mAb of DM1A (1:2000) were purchased from Sigma (St. Louis, MO, USA). DL-TBOA (30 *μ*M) and memantine (1 *μ*M) were from Tocris Bioscience (Bristol, UK). Neurobasal and B27 were from Invitrogen (Grand Island, NY, USA). PAb R134d (1:1000) against total tau was a generous gift from Dr K. Iqbal (New York State Institute for Basic Research, Staten Island, NY, USA).

### Animals and treatment

Tau Ko mice (4–5 months, male) were generated by crossing the human tau transgenic mice [B6.Cg-*Mapt*^*tm1(GFP)Klt*^ Tg(MAPT)8cPdav/J] from Jackson Lab (Bar Harbor, ME, USA). In these mice, endogenous *tau* gene was replaced by *EGFP* gene, while human tau failed to be expressed.^18^ The genotype was confirmed by PCR analysis of tail biopsies. Age-matched wild-type C57 male mice were from Experimental Animal Center of Tongji Medical College. All mice were kept under standard laboratory conditions: 12 h light and 12 h dark with water and food *ad libitum*. All animal experiments were performed according to the 'Policies on the Use of Animals and Humans in Neuroscience Research' revised and approved by the Society for Neuroscience in 1995.

For E-NMDAR activation *in vivo,* mice were deeply anesthetized, two holes were made for injection at coordinates 1.9 mm anterior to posterior bregma, 1.9 mm mid to lateral,^36^ 2.4 mm dorsal to ventral according to the stereotaxic atlas. About 2 *μ*l (60 mM) NMDA or saline was injected bilaterally into hippocampus of the mice. Twenty-four hours after the injection, mice were killed. The right half of the hippocampus was homogenized in 10 volumes (ml/g wet tissue) homogenate buffer containing 50 mM Tris-HCl, pH 7.0, 0.5 mM phenylmethanesulfonyl fluoride, 2.5 mM ethylene diamine tetraacetic acid, 2.5 mM ethylene glycol tetraacetic acid and 1:1000 protease inhibitor cocktail (Sigma-Aldrich, St. Louis, MO, USA). The other half was fixed in 4% paraformaldehyde for 48 h, then the half brains were washed in phosphate buffer (0.1 M, pH 7.4), immersed into 30% sucrose in phosphate buffer until the brains sink to the bottom. Then the samples were embedded in optimum cutting temperature compound, frozen and sectioned at 20 *μ*m using freezing microtome (Leica, Wetzlar, Germany).

### Primary neuronal culture and fluorescence imaging

Primary cortical neurons were isolated from embryonic E18 Sprague–Dawley rats or newly born wild-type and tau Ko mice as previously reported.^37^ Briefly, tissues were dissected, dissociated and incubated with 5 ml of D-Hanks containing 0.25% trypsin for 5 min, centrifuged at 1000 × *g* for 5 min after addition of 4 ml of the neuronal plating medium containing DMEM/F12 with 10% fetal bovine serum. Then the cells were resuspended, about 5 × 10^5^ cells were plated onto each well of 12-well plates for western blotting, and 1 × 10^5^ cells were plated onto each glass cover slip for cell imaging. Both the plates and the glass cover slips were previously coated with poly-d-lysine. The neurons were then put into a humidified incubator with 5% CO_2_ at 37 °C. The medium was changed to neurobasal medium supplemented with 2% B27 (maintenance medium) after 2–4 h. Cells were cultured for 12–14 days before treatment. During the culture, the medium was half-changed every 3 days with fresh maintenance medium.

For immunofluorescence, at the end of treatment, neurons were quickly fixed with 4% paraformaldehyde for 15 min, permeabilized in 0.1% Triton X-100 for 15 min, followed by incubation with 3% bovine serum albumin to block non-specific sites. Primary antibody incubation was performed overnight at 4 °C. Alexa 488- or 543-conjugated secondary antibody (1:1000) was used for fluorescence labeling. To visualize the neuronal morphology, EGFP was transfected into rat neurons at 11 DIV, 48 h later, neurons were treated with synaptic or extrasynaptic NMDAR activation protocols for 24 h. At the end of treatment, cells were fixed with 4% paraformaldehyde for fluorescence microscopy. For DAB staining, the immunoreaction was detected using horseradish peroxidase-labeled antibodies for 1 h at 37 °C. The imaging was observed with the LSM710 confocal microscope (Zeiss, Oberkochen, Germany).

For the morphological analysis of the wild-type neurons, primary mouse cortical neuron cultures (5 DIV) were infected by lentivirus to express EGFP. One week later, the morphology of the tau Ko mouse neurons (12 DIV) was observed directly under the fluorescence microscope as tau Ko neurons were genetically EGFP expressed.

### Western blotting and co-immunoprecipitation

For western blotting, samples were boiled at 100 °C for 5 min in the loading buffer (50 mM Tris-HCl, pH 7.6, 2% SDS, 10% glycerol, 10 mM DTT and 0.2% bromophenol blue). The proteins were electrophoresed in 10% SDS-PAGE and the separated proteins transferred to nitrocellulose membranes (Amersham Biosciences, Pittsburgh, USA). The membranes were then blocked with 5% non-fat milk dissolved in TBS-Tween-20 (50 mM Tris-HCl, pH 7.6, 150 mM NaCl, 0.2% Tween-20) for 1 h and probed with primary antibody at 4 °C overnight. Then the blots were detected using anti-rabbit or anti-mouse IgG conjugated to IRDye (800CW; Licor Biosciences, Lincoln, NE, USA) for 1 h at room temperature and visualized using the Odyssey Infrared Imaging System (Licor Biosciences). The protein bands were quantitatively analyzed by Kodak Digital Science 1D software (Eastman Kodak Company, New Haven, CT, USA).

To analyze protein–protein interactions, co-immunoprecipitation experiments were performed using wild-type or tau Ko mice brain homogenates. Specified antibody and protein G agarose were incubated with the homogenates overnight at 4 °C. The resins were washed for three times with PBS. After elution by 2 × loading buffer, and boiled at 95 °C for 5 min, the bound proteins were analyzed by western blotting.

### Real-time quantitative PCR

Total RNA was isolated using TRIzol reagents according to the instruction of the manufacturer (Invitrogen, Carlsbad, CA, USA) and reverse-transcribed to cDNA using reverse transcription reagents kit (Takara, Dalian, China). Fifty nanograms of cDNA were used for real-time PCR. Primers for rat tau: forward primer 5′-GGGACATGGGTGATGTTATCCAA-3′, reverse primer 5′-CCTGAGCAAGGTGACCTCCAA-3′ *β*-actin: forward primer 5′-GGAGATTACTGCCCTGGCTCCTA-3′, reverse primer 5′-GACTCATCGTACTCCTGCTTGCTG-3′. The PCR cycle was as follows: 95 °C/30 s, 40 cycles of 95 °C/5 s, 60 °C/30 s and 72 °C/30 s, and the melt-curve analysis was performed following each experiment. The amplification and analysis were performed using a StepOnePlus Real-Time PCR Detection System (Thermo Fisher, New York, NY, USA). Samples were compared using the relative CT method.

### LDH assay

Wild-type or tau Ko mouse primary cortical neurons were cultured in 96-well plates (about 1 × 10^4^ cells per well) for 12 days. Cells were then subject to extrasynaptic NMDAR activation. Cytotoxicity was determined by LDH assay 12 or 24 h after the treatment. The LDH assay was performed using Pierce LDH Cytotoxicity Assay Kit (Pierce, Rockford, USA) according to the manufacturer's instructions.

### Quantification of neuronal cell apoptosis

To evaluate neuronal apoptosis triggered by extrasynaptic NMDAR activation, wild-type and tau Ko mouse primary neurons were cultured for 12 days and extrasynaptic NMDARs were activated for 24 h. Hoechst 33342, a blue fluorescence dye was used to stain the neurons. Hoechst reagent stains the condensed chromatin in apoptotic cells brightly.

### Nissl staining

The brain slices were soaked in 1% toluidine blue for 3 min. Sections were then dehydrated using 95 and 100% ethanol solutions, transparented using xylene, placed under cover slips and analyzed with a microscope (Nikon, 90i, Tokyo, Japan).

### PP2A activity assay

PP2A activity was measured according to the protocol provided by the manufacturer (V2460 kit, Promega, Madison, USA). First, endogenous-free phosphate was removed from the wild-type or tau Ko C57 brain homogenates, and then the extracts were normalized for protein content. About 5 *μ*g of protein samples in triplicates were incubated with a chemically synthesized phosphopeptide (RRA(pT)VA), an optimal substrate for PP2A, PP2B and PP2C, not for PP1 in the buffer optimized for PP2A activity while cation-dependent PP2B and PP2C were inhibited for 30 min at 33 °C. Phosphate released from the substrate was detected by measuring the absorbance of a molybdate-malachite green-phosphate complex at 630 nm. PP2A activity was calculated by the release of phosphate per *μ*g of protein and per minute (pmol/*μ*g/min).

For ERK-related PP2A activity assays, ERK antibody and protein G were incubated with the homogenates overnight at 4 °C. The resins were washed three times with PBS. After elution by the buffer, PP2A activity was detected using the method above.

### *In vitro* ERK dephosphorylation assay

*In vitro* ERK dephosphorylation experiment was performed in wild-type and tau Ko brain homogenates. Briefly, 50 ng of purified phosphorylated His-tagged ERK protein was added to 50 *μ*g homogenates, and was incubated at room temperature for 10 min in the presence of MEK inhibitor U0126 (10 *μ*M). Another part of tau Ko mice brain homogenates was pre-incubated with purified 100 ng recombinant human Tau441 proteins. At the end of incubation, anti-His-tag antibody and protein G agarose were added and incubated overnight at 4 °C. The resins were washed for three times with PBS. After elution by 2 × loading buffer, and boiled at 95 °C for 5 min, the bound proteins were analyzed for p-ERK and total ERK expression by western blotting.

### Statistical analysis

Data are expressed as mean±S.D. and analyzed using SPSS 16.0 statistical software (SPSS Inc., Chicago, IL, USA). The one-way analysis of variance (ANOVA) procedure followed by LSD's *post hoc* tests was used to determine the differences among groups. The significance was assessed at *P*<0.05. All results shown correspond to individual representative experiments.

## Abbreviations

NFTs: neurofibrillary tangles
AD: Alzheimer disease
NMDA: N-Methyl-d-aspartate
NMDAR: NMDA receptor
E-NMDAR: extrasynaptic NMDA receptor
Bic: bicucullin
4-AP: 4-aminopyridine
DL-TBOA: Dl-threo-b-benzyloxyaspartic acid
Wt: wild type, tau Ko: tau knockout
ERK: extracellular signal-regulated kinase
MEK: mitogen-activated and extracellular signal-regulated kinase kinase
PTP1B: protein tyrosine phosphatase-1B
PP2A: protein phosphotase 2 A
PKA: protein kinase A
CREB: CAMP response element binding protein
DAB staining: diaminobenzidine staining
LDH assay: lactate dehydrogenase assay
EGFP: enhanced green fluorescent protein

## References

[bib1] Khatoon S, Grundke-Iqbal I, Iqbal K. Levels of normal and abnormally phosphorylated tau in different cellular and regional compartments of Alzheimer disease and control brains. FEBS Lett 1994; 351: 80–84. 8076698 10.1016/0014-5793(94)00829-9

[bib2] Khatoon S, Grundke-Iqbal I, Iqbal K. Brain levels of microtubule-associated protein tau are elevated in Alzheimer's disease: a radioimmuno-slot-blot assay for nanograms of the protein. J Neurochem 1992; 59: 750–753. 1629745 10.1111/j.1471-4159.1992.tb09432.x

[bib3] Barton AJ, Harrison PJ, Najlerahim A, Heffernan J, McDonald B, Robinson JR et al. Increased tau messenger RNA in Alzheimer's disease hippocampus. Am J Pathol 1990; 137: 497–502. 2119143 PMC1877517

[bib4] Yasojima K, McGeer EG, McGeer PL. Tangled areas of Alzheimer brain have upregulated levels of exon 10 containing tau mRNA. Brain Res 1999; 831: 301–305. 10412011 10.1016/s0006-8993(99)01486-9

[bib5] Obulesu M, Venu R, Somashekhar R. Tau mediated neurodegeneration: an insight into Alzheimer's disease pathology. Neurochem Res 2011; 36: 1329–1335. 21509508 10.1007/s11064-011-0475-5

[bib6] Pooler AM, Noble W, Hanger DP. A role for tau at the synapse in Alzheimer's disease pathogenesis. Neuropharmacology 2014; 76(Pt A): 1–8. 24076336 10.1016/j.neuropharm.2013.09.018

[bib7] Bliss TV, Collingridge GL. A synaptic model of memory: long-term potentiation in the hippocampus. Nature 1993; 361: 31–39. 8421494 10.1038/361031a0

[bib8] Hardingham GE, Fukunaga Y, Bading H. Extrasynaptic NMDARs oppose synaptic NMDARs by triggering CREB shut-off and cell death pathways. Nat Neurosci 2002; 5: 405–414. 11953750 10.1038/nn835

[bib9] Milnerwood AJ, Gladding CM, Pouladi MA, Kaufman AM, Hines RM, Boyd JD et al. Early increase in extrasynaptic NMDA receptor signaling and expression contributes to phenotype onset in Huntington's disease mice. Neuron 2010; 65: 178–190. 20152125 10.1016/j.neuron.2010.01.008

[bib10] Tu W, Xu X, Peng L, Zhong X, Zhang W, Soundarapandian MM et al. DAPK1 interaction with NMDA receptor NR2B subunits mediates brain damage in stroke. Cell 2010; 140: 222–234. 20141836 10.1016/j.cell.2009.12.055 PMC2820131

[bib11] Bordji K, Becerril-Ortega J, Nicole O, Buisson A. Activation of extrasynaptic, but not synaptic, NMDA receptors modifies amyloid precursor protein expression pattern and increases amyloid-ss production. J Neurosci 2010; 30: 15927–15942. 21106831 10.1523/JNEUROSCI.3021-10.2010 PMC6633754

[bib12] Leveille F, El Gaamouch F, Gouix E, Lecocq M, Lobner D, Nicole O et al. Neuronal viability is controlled by a functional relation between synaptic and extrasynaptic NMDA receptors. FASEB J 2008; 22: 4258–4271. 18711223 10.1096/fj.08-107268

[bib13] Esclaire F, Lesort M, Blanchard C, Hugon J. Glutamate toxicity enhances tau gene expression in neuronal cultures. J Neurosci Res 1997; 49: 309–318. 9260742 10.1002/(sici)1097-4547(19970801)49:3<309::aid-jnr6>3.0.co;2-g

[bib14] Leveille F, Papadia S, Fricker M, Bell KF, Soriano FX, Martel MA et al. Suppression of the intrinsic apoptosis pathway by synaptic activity. J Neurosci 2010; 30: 2623–2635. 20164347 10.1523/JNEUROSCI.5115-09.2010 PMC2834927

[bib15] Tatebayashi Y, Haque N, Tung YC, Iqbal K, Grundke-Iqbal I. Role of tau phosphorylation by glycogen synthase kinase-3beta in the regulation of organelle transport. J Cell Sci 2004; 117(Pt 9): 1653–1663. 15075227 10.1242/jcs.01018

[bib16] Chalifoux JR, Carter AG. Glutamate spillover promotes the generation of NMDA spikes. J Neurosci 2011; 31: 16435–16446. 22072693 10.1523/JNEUROSCI.2777-11.2011 PMC3235338

[bib17] Lipton SA. The molecular basis of memantine action in Alzheimer's disease and other neurologic disorders: low-affinity, uncompetitive antagonism. Curr Alzheimer Res 2005; 2: 155–165. 15974913 10.2174/1567205053585846

[bib18] Tucker KL, Meyer M, Barde YA. Neurotrophins are required for nerve growth during development. Nat Neurosci 2001; 4: 29–37. 11135642 10.1038/82868

[bib19] Nagendra SN, Faiman MD, Davis K, Wu JY, Newby X, Schloss JV. Carbamoylation of brain glutamate receptors by a disulfiram metabolite. J Biol Chem 1997; 272: 24247–24251. 9305877 10.1074/jbc.272.39.24247

[bib20] Chung KC, Shin SW, Yoo M, Lee MY, Lee HW, Choe BK et al. A systemic administration of NMDA induces immediate early gene pip92 in the hippocampus. J Neurochem 2000; 75: 9–17. 10854241 10.1046/j.1471-4159.2000.0750009.x

[bib21] Zhou B, Wang ZX, Zhao Y, Brautigan DL, Zhang ZY. The specificity of extracellular signal-regulated kinase 2 dephosphorylation by protein phosphatases. J Biol Chem 2002; 277: 31818–31825. 12082107 10.1074/jbc.M203969200

[bib22] Fukukawa C, Tanuma N, Okada T, Kikuchi K, Shima H. pp32/ I-1(PP2 A) negatively regulates the Raf-1/MEK/ERK pathway. Cancer Lett 2005; 226: 155–160. 16039954 10.1016/j.canlet.2004.11.026

[bib23] Gendreau KL, Hall GF. Tangles, toxicity, and Tau secretion in AD - new approaches to a vexing problem. Front Neurol 2013; 4: 160. 24151487 10.3389/fneur.2013.00160 PMC3801151

[bib24] Chen HS, Wang YF, Rayudu PV, Edgecomb P, Neill JC, Segal MM et al. Neuroprotective concentrations of the N-methyl-D-aspartate open-channel blocker memantine are effective without cytoplasmic vacuolation following post-ischemic administration and do not block maze learning or long-term potentiation. Neuroscience 1998; 86: 1121–1132. 9697119 10.1016/s0306-4522(98)00163-8

[bib25] Lipton SA. Paradigm shift in NMDA receptor antagonist drug development: molecular mechanism of uncompetitive inhibition by memantine in the treatment of Alzheimer's disease and other neurologic disorders. J Alzheimers Dis 2004; 6(6 Suppl): S61–S74. 15665416 10.3233/jad-2004-6s610

[bib26] Parsons CG, Stoffler A, Danysz W. Memantine: a NMDA receptor antagonist that improves memory by restoration of homeostasis in the glutamatergic system—too little activation is bad, too much is even worse. Neuropharmacology 2007; 53: 699–723. 17904591 10.1016/j.neuropharm.2007.07.013

[bib27] Liu H, Jin X, Yin X, Jin N, Liu F, Qian W. PKA-CREB signaling suppresses Tau transcription. J Alzheimers Dis 2015; 46: 239–248. 25720403 10.3233/JAD-142610

[bib28] Vanhoutte P, Bading H. Opposing roles of synaptic and extrasynaptic NMDA receptors in neuronal calcium signalling and BDNF gene regulation. Curr Opin Neurobiol 2003; 13: 366–371. 12850222 10.1016/s0959-4388(03)00073-4

[bib29] Li HL, Wang HH, Liu SJ, Deng YQ, Zhang YJ, Tian Q et al. Phosphorylation of tau antagonizes apoptosis by stabilizing beta-catenin, a mechanism involved in Alzheimer's neurodegeneration. Proc Natl Acad Sci USA 2007; 104: 3591–3596. 17360687 10.1073/pnas.0609303104 PMC1805527

[bib30] Frandemiche ML, De Seranno S, Rush T, Borel E, Elie A, Arnal I et al. Activity-dependent tau protein translocation to excitatory synapse is disrupted by exposure to amyloid-beta oligomers. J Neurosci 2014; 34: 6084–6097. 24760868 10.1523/JNEUROSCI.4261-13.2014 PMC6608293

[bib31] Liang Z, Liu F, Iqbal K, Grundke-Iqbal I, Gong CX. Dysregulation of tau phosphorylation in mouse brain during excitotoxic damage. J Alzheimers Dis 2009; 17: 531–539. 19363259 10.3233/JAD-2009-1069 PMC2829309

[bib32] Tian FF, Guo TH, Dang J, Ma YF, Chen JM, Chen Y et al. [Involvement of Cdk5/p35 and tau protein in the hippocampal mossy fiber sprouting in the PTZ kindling model]. Zhonghua Yi Xue Za Zhi 2011; 91: 1197–1202. 21756775

[bib33] Ittner LM, Ke YD, Delerue F, Bi M, Gladbach A, van Eersel J et al. Dendritic function of tau mediates amyloid-beta toxicity in Alzheimer's disease mouse models. Cell 2010; 142: 387–397. 20655099 10.1016/j.cell.2010.06.036

[bib34] Amadoro G, Ciotti MT, Costanzi M, Cestari V, Calissano P, Canu N. NMDA receptor mediates tau-induced neurotoxicity by calpain and ERK/MAPK activation. Proc Natl Acad Sci USA 2006; 103: 2892–2897. 16477009 10.1073/pnas.0511065103 PMC1413822

[bib35] Subramaniam S, Unsicker K. ERK and cell death: ERK1/2 in neuronal death. FEBS J 2010; 277: 22–29. 19843173 10.1111/j.1742-4658.2009.07367.x

[bib36] Yu LG, Packman LC, Weldon M, Hamlett J, Rhodes JM. Protein phosphatase 2 A, a negative regulator of the ERK signaling pathway, is activated by tyrosine phosphorylation of putative HLA class II-associated protein I (PHAPI)/pp32 in response to the antiproliferative lectin, jacalin. J Biol Chem 2004; 279: 41377–41383. 15247276 10.1074/jbc.M400017200

[bib37] Sun XY, Wei YP, Xiong Y, Wang XC, Xie AJ, Wang XL et al. Synaptic released zinc promotes tau hyperphosphorylation by inhibition of protein phosphatase 2A (PP2A). J Biol Chem 2012; 287: 11174–11182. 22334661 10.1074/jbc.M111.309070 PMC3322889

