# Supplementary material for: Extrasynaptic NMDA receptor-induced tau overexpression mediates neuronal death through suppressing survival signaling ERK phosphorylation
Source: Cell Death Dis. 2016 Nov 3;7(11):e2449–. doi: 10.1038/cddis.2016.329 (PMC5260900; doi:10.1038/cddis.2016.329)
Supplement: Supplementary Information [file cddis2016329x1.doc]

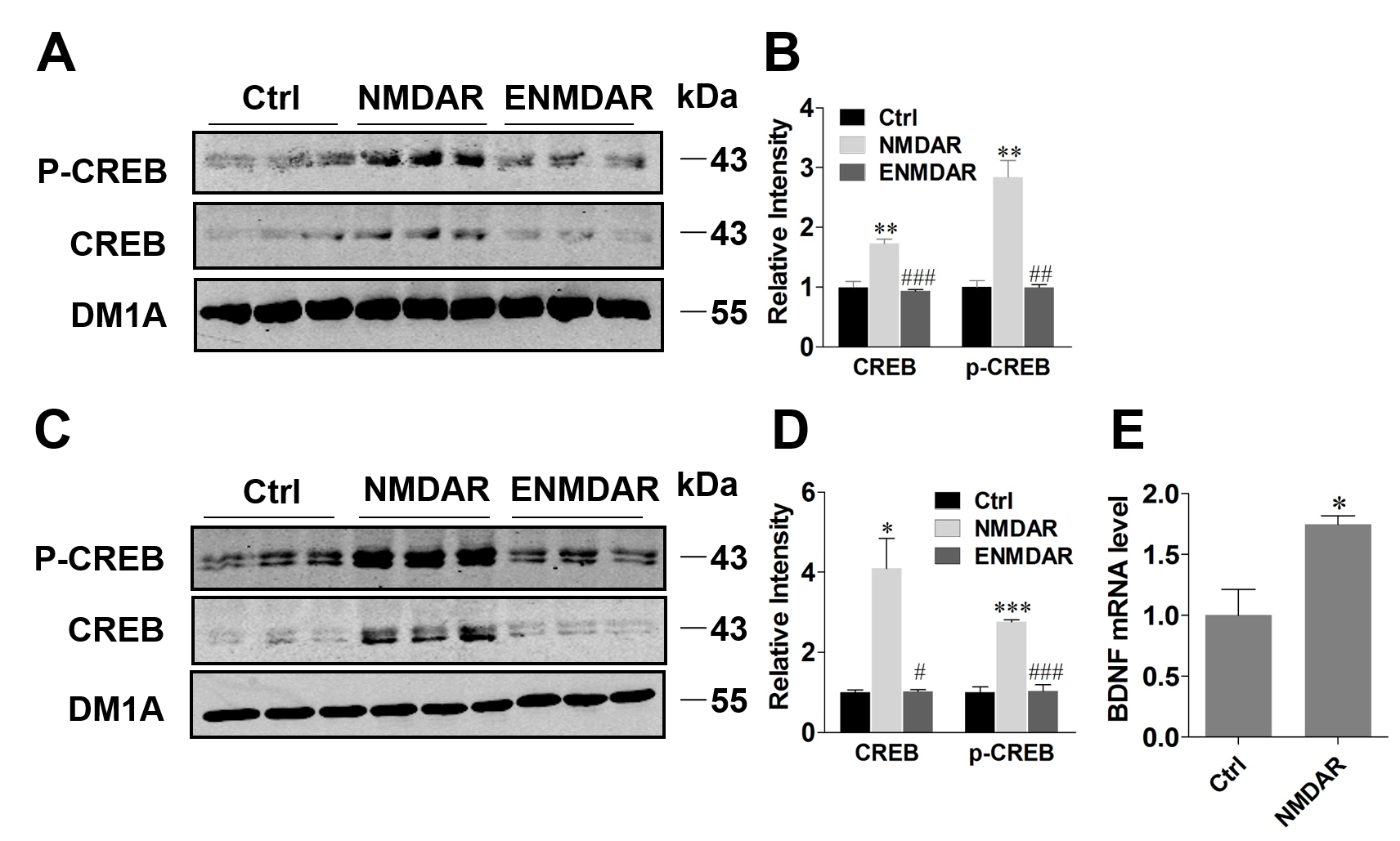


**Supplementary Figure 1- Activation of synaptic or extrasynaptic NMDA receptors by used protocols.** Rat primary cortical neurons (12-14 DIV) were incubated with Bic (50 μM) /4-AP (2.5 mM) to activate synaptic NMDA receptors for 12 or 24 h (herein using NMDAR for abbreviation). To specifically induce extrasynaptic NMDA receptors (E-NMDAR) activation, neurons were incubated with Bic (50 μM) /4-AP (2.5 mM) for 2 min, after wash, open NMDA receptors blocker MK-801 (10 μM) was administrated for another 2 min to block synaptic NMDA receptors, at last NMDA (30 μM) and glycine (10 μM) were used to selectively activate E-NMDARs for 12 or 24 h (herein using ENMDAR for abbreviation). To confirm the activation of synaptic or extrasynaptic NMDA receptors, CREB and p-CREB were detected by Western blotting at 12 h (A, B) or 24 h (C, D). Synaptic NMDAR activation resulted in robust phosphorylation of CREB, with increased total CREB level, while CREB phosphorylation was shut off in ENMDAR group. CREB, p-CREB levels were normalized with DM1A, n = 5. BDNF mRNA levels were detected by q-PCR at 12 h (E). Synaptic activity induced significant increase of BDNF mRNA levels, which were normalized with β-actin mRNA levels. n = 5. * *p*<0.05, ** *p*<0.01, *** *p*<0.001 vs control neurons. # *p*<0.05, ## *p*<0.01, ### *p*<0.001 vs NMDAR neurons.


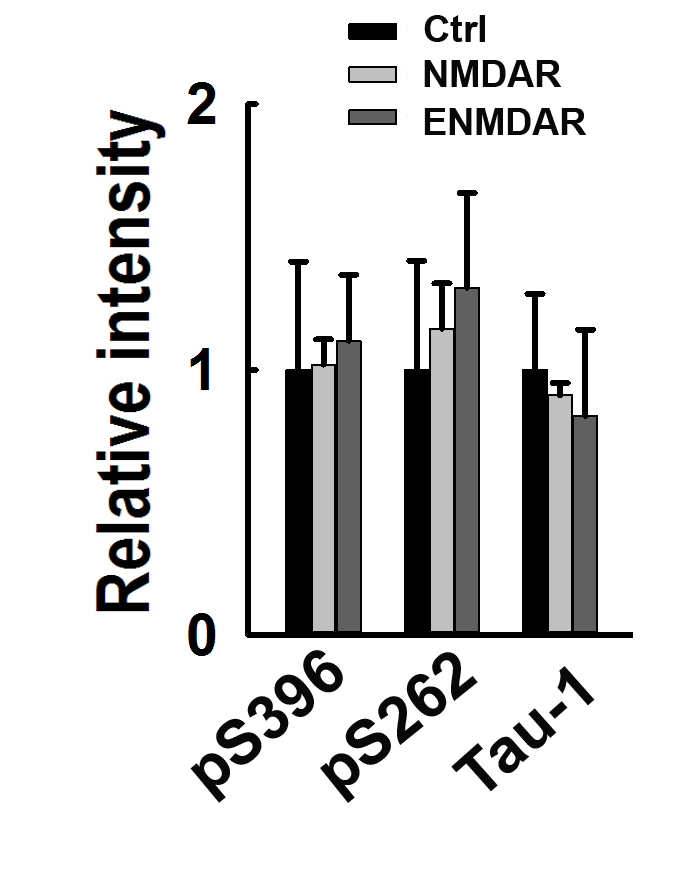


**Supplementary Figure 2- Normalization of tau phosphorylation and dephosphorylation levels by total tau.** Tau phosphorylation and dephosphorylation levels in Fig 1A (24 h) were evaluated by the ratio of pS396 and pS262 phosphorylated tau or Tau-1 dephosphorylated tau levels to total tau level (R134d).


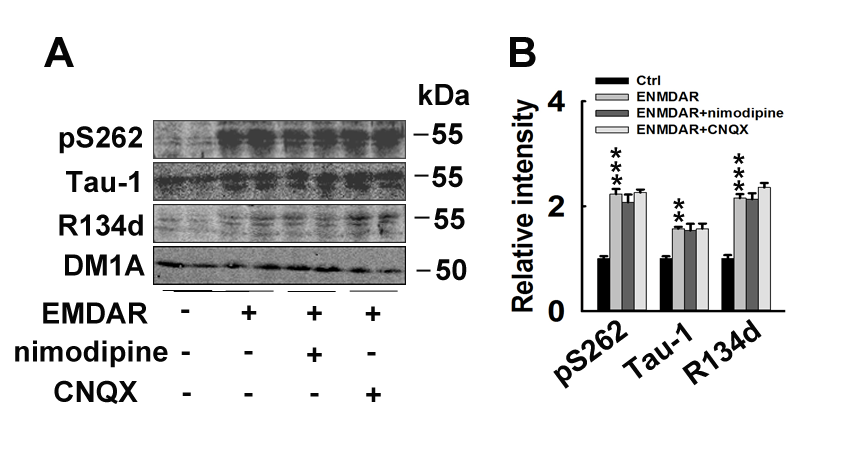


**Supplementary Figure 3- Voltage-sensitive calcium channel (VSCC) or AMPA/kainate receptor are not involved in tau overexpression induced by extrasynaptic NMDA receptors activation protocol.** (A) Primary cultured rat cortical neurons at DIV 12-14 were subjected to extrasynaptic NMDAR activation for 24 h with or without nimodipine (10 µM, VSCC blocker) or CNQX (10 µM, AMPA receptors antagonist) pre-incubation for half an hour. Total (R134d), phosphorylated (pS262) and dephosphorylated (Tau-1) tau levels were detected by Western blotting. (B) Quantitative analysis of the blots in (A). Total, phosphorylated and dephosphorylated tau levels were normalized with DM1A. ** p<0.01, *** p<0.001 vs control group; n = 6, N = 3 independent cultures.
